# Novel mutations in natriuretic peptide receptor-2 gene underlie acromesomelic dysplasia, type maroteaux

**DOI:** 10.1186/1471-2350-13-44

**Published:** 2012-06-12

**Authors:** Saadullah Khan, Raja Hussain Ali, Sanaullah Abbasi, Muhammad Nawaz, Noor Muhammad, Wasim Ahmad

**Affiliations:** 1 Department of Biochemistry, Faculty of Biological Sciences, Quaid-i-Azam University Islamabad, Islamabad, Pakistan; 2 Department of Biotechnology & Genetic Engineering, Kohat University of Science & Technology (KUST), Kohat, Pakistan

**Keywords:** Acromesomelic dysplasia-type Maroteaux, gene *NPR2*, missence mutation (T907M), splice site mutation c.2986 + 2 T > G

## Abstract

**Background:**

Natriuretic peptides (NPs) are peptide hormones that exert their biological actions by binding to three types of cell surface natriuretic peptide receptors (NPRs). The receptor NPR-B binding C-type natriuretic peptide (CNP) acts locally as a paracrine and/or autocrine regulator in a wide variety of tissues. Mutations in the gene *NPR2* have been shown to cause acromesomelic dysplasia-type Maroteaux (AMDM), an autosomal recessive skeletal disproportionate dwarfism disorder in humans.

**Methods:**

In the study, presented here, genotyping of six consanguineous families of Pakistani origin with AMDM was carried out using polymorphic microsatellite markers, which are closely linked to the gene *NPR2* on chromosome 9p21-p12. To screen for mutations in the gene *NPR2*, all of its coding exons and splice junction sites were PCR amplified from genomic DNA of affected and unaffected individuals of the families and sequenced.

**Results:**

Sequence analysis of the gene *NPR2* identified a novel missence mutation (p.T907M) in five families, and a splice donor site mutation c.2986 + 2 T > G in the other family.

**Conclusion:**

We have described two novel mutations in the gene *NPR2.* The presence of the same mutation (p.T907M) and haplotype in five families (A, B, C, D, E) is suggestive of a founder effect.

## Background

Acromesomelic Dysplasia, type Maroteaux (AMDM) is characterized by disproportionate shortening of skeletal elements, predominantly affecting the middle segments (forearms and forelegs) and distal segments (hands and feet) of appendicular skeleton. In addition, axial skeletal involvement occurs characterized by wedging of vertebral bodies, with dorsal margins being shorter than the ventral margins. Mode of inheritance of AMDM (MIM 602875) is an autosomal recessive with a prevalence of 1/1,000,000 [1]. Skeletal growth in AMDM patients falls off sharply after birth causing abnormal growth plate and short misshapen bones in the extremities and spine [2]. Carrier parents of AMDM children are shorter than average [2].

Acromesomelic dysplasia was mapped on chromosome 9p13-q12 [3]. Later, Bartels et al. [4] identified mutations in gene *NPR2*, encoding natriuretic peptide receptor B (NPR-B), underlying Acromesomelic Dysplasia, Type Maroteaux. Natriuretic peptides (NPs) comprise a family of hormones involved in the regulation of various physiological processes including cardiac growth, blood pressure, axonal path finding and endochondral ossification by binding to cell surface receptors called natriuretic peptide receptors (NPRs) [5-7]. Three different subtypes (A, B, C) of NPRs have been identified [8]. The NPR-B is expressed in various tissues and cell populations like heart, vessels, brain, uterus and chondrocytes [8-10]. NPR-B is a receptor for C-type natriuretic peptide (CNP) that acts locally as a paracrine and/or autocrine regulator in a wide variety of tissues [11]. NPR-B consists of an extracellular ligand binding domain, a single hydrophobic transmembrane region, an intracellular kinase homology domain (KHD), and carboxyl- terminal guanylyl cyclase (GC) domain [11,12]. The NPR-A and NPR-B mediate their biological function through GC domains.

In the report, presented here, we have investigated 6 consanguineous Pakistani families with multiple affected individuals showing typical features of acromesomelic dysplasia, Type Maroteaux. DNA sequence analysis of the gene *NPR2* detected a novel homozygous missense mutation (Thr907Met) in five families (A-E) and a novel splice site mutation c.2986 + 2 T > G [IVS20 + 2 T > G] in affected individuals of the sixth family (F).

## Methods

### Subjects and ethical approval of the study

**Figure 1:**
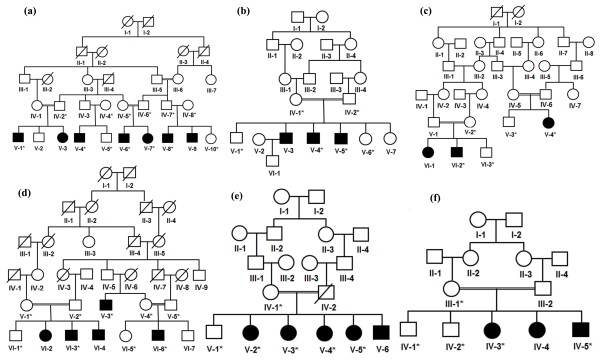
**Pedigrees of six consanguineous Pakistani families segregating autosomal recessive form of AMDM.** Double lines are indicative of consanguineous union. Clear symbols represent unaffected individuals while filled symbols represent affected individuals. The diagonal line through a symbol is indicative of a deceased family member. Symbols with asterisks indicate individuals who were clinically examined and for whom DNA samples were available for molecular analysis.

The present study presents six consanguineous families (A, B, C, D, E, F) of Pakistani origin exhibiting autosomal recessive form of acromesomelic dysplasia, type Maroteaux (AMDM). Five families (A, B, C, D, E) were ascertained from Punjab province and family F from Sindh province of Pakistan. Forty four individuals including 18 affected volunteered to participate in the study (Figure 1). The study was conducted after obtaining informed consent from the patients and their parents, and permission to undertake the study was obtained from the institutional review board (IRB) of Quaid-i-Azam University, Islamabad Pakistan.

### DNA isolation and genotyping

Blood samples from both affected and unaffected individuals of the six families were collected in EDTA containing vacutainer sets (BD, Franklin NG, USA). Genomic DNA from the blood samples was extracted using GenElute^TM^ Blood Genomic DNA Kit (Sigma-Aldrich, MO, USA). To quantify DNA, Nanodrop-1000 spectrophotometer (Thermal Scientific, Wilmington, USA) was used, measuring optical density at 260 nm and diluted to 40–50 ng/μl for amplification by polymerase chain reaction (PCR). PCR amplifications conditions for microsatellite markers were the same as described earlier by Khan et al. [13]. Allele size for respective microsatellite markers were determined using 05-bp, 10-bp and 20-bp DNA ladders (MBI, Fermentas®, York, UK).

Previously, it has been reported that mutations in the gene *NPR2* result in acromesomelic dysplasia, type Maroteaux (AMDM). Therefore, linkage in the six families was tested by genotyping microsatellite markers (D9S1118, D9S1845, D9S1817, D9S50, D9S1874) linked to the gene *NPR2* mapped on chromosome 9p13-q12.

### Screening *NPR2*

All 22 exons of *NPR2* with adjacent sequences of exon-intron borders were amplified by PCR using gene specific primers. The amplification conditions used were 95^0^ C for 1 minute, followed by 30 cycles of 95^0^ C for 35 seconds, 60^0^ C for 35 seconds, and 70^0^ C for 3.5 minutes, followed by a single incubation at 70^0^ C for 10 minutes. The PCR products were purified using the Rapid PCR Purification System 9700 (Marligen Biosciences, Ijamsville, MD, USA) and sequenced bidirectionally (both forward and reverse) following dideoxy chain termination method using DTCS Quick Start sequencing kit on CEQ8800 DNA sequencer (Beckman Coulter, Inc. Brea, CA USA) according to the manufacturer's instructions. Sequence variants were identified via BioEdit sequence alignment editor version 6.0.7 (http://www.mbio.ncsu.edu/BioEdit/bioedit.html). PCR primers were designed using the ‘Primer3’ program (http://frodo.wi.mit.edu/primer3) [14] and checked for specificity using basic local alignment search tool (BLAST; http://www.ncbi.nlm.nih.gov/blast). The possible impact of amino acid substitution on the structure of the NPR-B protein was examined with PolyPhen2 tools (http://genetics.bwh.harvard.edu/pph2). Evolutionary conservation of the mutated amino acid threonine in NPR-B in orthologs was examined using http://www.ncbi.nlm.nih.gov/homologene/.

## Results

### Clinical features

**Figure 2:**
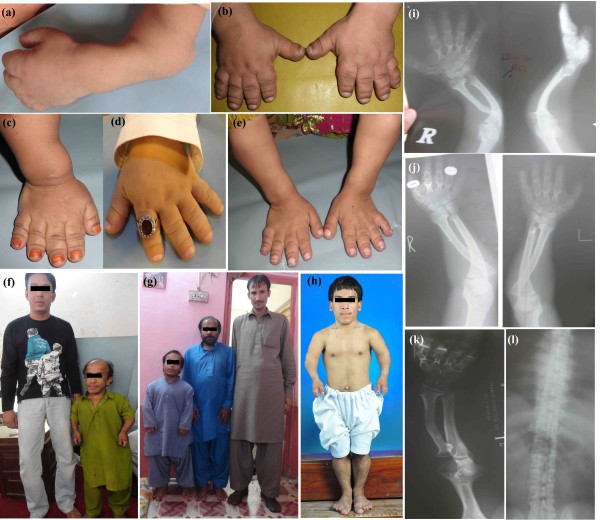
**Clinical features of AMDM.** Bowing of forearm in an affected member **(**V-8**)** in family A **(a)**, affected members including V-5 in family B, V-4 in family C, VI-6 in family D, V-2 in family E showing short fingers and redundant skin **(b-e)**, an affected member V-4 at 38 years of age with his younger brother V-5 at 27 years of age in family A **(f)**, an affected member V-3 at 36 years of age with a carrier VI-1 at 29 years of age in family D along with one of the authors **(g)**, an affected member IV-5 in family F showing short stature and short extremities **(h)**. Radiograph of hand and forearm of an affected member V-4 in family A **(i)**, V-3 in family D (j), IV-5 of family F **(k)**, showing epiphysis of the radius, shortening of ulna, short and stubby metacarpels. Radiograph of vertebral column of an affected member IV-5 in family F showing mild platyspondyly **(l).**

Affected individuals in all six families exhibited features of Acromesomelic Dysplasia, Type Maroteaux (AMDM). Ages of the affected members ranged from 9–38 years at the time of the study. Table 1 lists heights of the affected individuals of the families. Mostly similar clinical features were observed in affected members of all the six families (Table 2, Figure 2a-e). However, affected individuals in family F had long faces (Figure 2h). Fingers of the affected members were extremely short with redundant skin. Limbs showed marked shortening in the middle and distal segments. A skeletal survey revealed disproportionate mesomelic shortening of the arms, phalanges and metacarpal bones. Sterility was not observed in patients of all the six families, presented here.

**Table 1:** Comparison of heights of individuals affected with AMDM, heterozygous parents and control Pakistani population

**Family**	**Affected Member**	**Sex**	**Age (Years)**	**Height (cm)**	**Father Height (cm)**	**Mother Height (cm)**
					**(Average Matched Control Height of Pakistani Population)**	
A	V-I	M	27	118	158.5 (164.0)	NA (152.5)
A	V-4	M	38	128	NA (164.0)	147.0 (152.5)
A	V-6	M	24	105	155.0 (164.0)	145.5 (152.5)
A	V-7	F	17	101	155.0 (164.0)	145.5 (152.5)
B	V-2	M	11	89	158.0 (164.0)	146.0 (152.5)
C	V-4	F	22	103	NA (164.0)	NA (152.5)
C	VI-2	M	18	104	NA (164.0)	145.0 (152.5)
D	V-3	M	36	127	NA (164.0)	NA (152.5)
D	VI-3	M	26	113	153.0 (164.0)	147.0 (152.5)
D	VI-6	M	9	73	157.0 (164.0)	146.5 (152.5)
E	V-2	F	20	102	NA (164.0)	144.5 (152.5)
E	V-3	F	17	96	NA (164.0)	144.5 (152.5)
E	V-4	F	14	91	NA (164.0)	144.5 (152.5)
E	V-5	F	12	82	NA (164.0)	144.5 (152.5)
F	IV-3	F	24	109	NA (164.0)	147.5 (152.5)
F	IV-5	M	21	113	NA (164.0)	147.5 (152.5)

NA, not available.

**Table 2:** Skeletal malformations observed in families with AMDM

**Family**	**Organs**	**Clinical Features**	**Radiological Features**
A	Long bones	Short middle and distal segments	Ulna shorter than radius, bilateral triangular distal epiphysis and bowing of the radius
	Hands and feet	Short and broad fingers with redundant skin	Short and broad phalanges. Short and stubby metacarpal and metatarsal bones.
	Vertebral abnormalities		Mild reduction in the heights of the vertebral bodies in the thoracic and lumbar spine (Mild platyspondyly)
B	Long bones	Short middle and distal segments	Ulna shorter than radius, bowing of the radius
	Hands and feet	Extremely short and broad fingers with redundant skin	Short and broad phalanges, metacarpal and metatarsal bones
	Vertebral abnormalities		Not available
C	Long bones	Short and misshaped middle and distal segments	Not available
	Hands and feet	Extremely short and broad fingers with redundant skin, great toe relatively large	Not available
	Vertebral abnormalities		Not available
D	Long bones	Short middle and distal segments	Ulna shorter than radius, bilateral triangular distal epiphysis and bowing of the radius
	Hands and feet	Short and broad fingers with redundant skin	Short and broad phalanges, metacarpal and metatarsal bones
	Vertebral abnormalities		Reduction in the heights in the thoracic and lumbar spine (Mild platyspondyly)
E	Long bones	Short and misshaped middle and distal segments	Not available
	Hands and feet	Short and broad fingers with slight redundant skin	Not available
	Vertebral abnormalities		Not available
F	Long bones	Short and misshaped middle and distal segments	Ulna shorter than radius, bilateral triangular distal appearance of epiphysis and bowing of the radius
	Hands and feet	Short and broad fingers with redundant skin, great toe relatively large	Short and broad phalanges, metacarpal and metatarsal bones
	Vertebral abnormalities		Mild reduction in the heights of the vertebral bodies in the thoracic and lumbar spine (Mild platyspondyly)
	Face	Long face	

Radiographs of affected individuals (V-4 of family A, V-3 of family D, IV-5 of family F) showed bilateral triangular distal epiphysis of the radius and relative shortening of the ulna. Metacarpels were short and stubby bilaterally. Mild reduction in the heights of vertebral bodies was noted in the thoracic and lumbar spine (mild platyspondyly).

### Linkage and mutation analysis

Genotyping data and haplotype analysis showed linkage of all the six families to the gene *NPR2* mapped on chromosome 9p13-q12. Subsequently, the gene *NPR2* was sequenced in two affected and one unaffected individual of each of the six families. Upon identifying the sequence variants, the same exon of the gene was sequenced in rest of the affected and unaffected individuals of the respective family. Sequence analysis of the gene *NPR2* was performed using a control reference obtained from the Ensembl database (ENSG00000159899).

**Figure 3:**
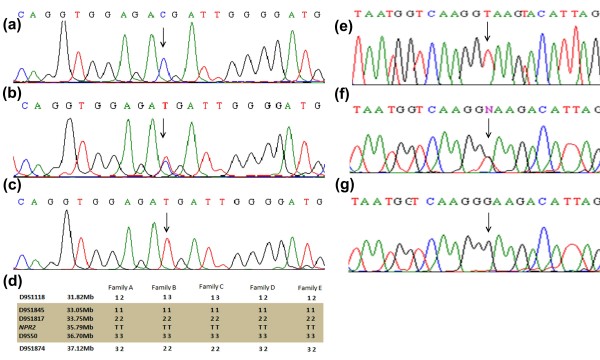
**Sequence analysis of the two novel mutations including c.2027 C > T (p.Thr907Met) identified in the gene*NPR2*in the five families (A-E), and c.2986 + 2 T > G [IVS20 + 2 T > G] in family F.** The upper panels **(a, e)** represent the nucleotide sequences in the unaffected individuals, the middle panels **(b, f)** in the heterozygous carriers and the lower panels **(c, g)** in the affected individuals. Panel d shows shared haplotypes of the gene *NPR2* linked microsatellite markers in affected individuals in five families (A-E). Disease-interval is flanked by markers D9S1118 and D9S1874. Arrows in the panels indicate position of the nucleotide change.

**Figure 4:**
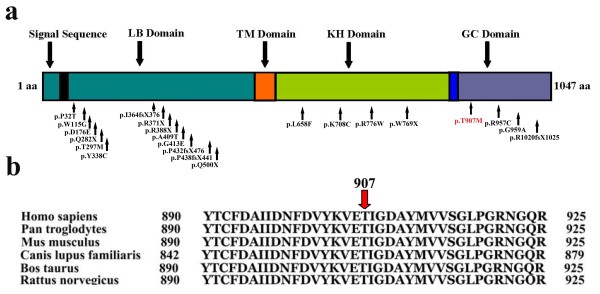
**a. Schematic representation of the human NPR-B structural and functional domains. Position of the missense mutation (p.T907Met) identified in NPR-B in the five families (A-E) is indicated by an arrow.** LB, Ligand Binding: TM, Trans Membrane; KH, Kinase Homology; GC, Guanylyl Cyclase; aa, amino acid. **b.** Partial amino acid sequence comparison of human NPR-B protein with other orthologs. An arrow indicates threonine (T) residue conserved across different species.

Sequence analysis of the gene *NPR2* detected a homozygous C to T transition at nucleotide position 2720 (c.2720 C > T) in affected individuals of the five families (A, B, C, D, E) (Figure 3a, b, c). This sequence change resulted in substitution of a threonine residue with methionine at amino acid position 907 (p.T907M) in the NPR-B protein (Figure 4). Upon examining the haplotypes, it was observed that a missense mutation (c.2720 C > T; p.T907M) in the families A-E appeared on very similar haplotypes, suggesting that the mutation in these five families was due to single mutation event (Figure 3d).

In family F, sequence analysis detected a splice donor site mutation in intron 20 of the gene (c.2986 + 2 T > G) (Figure 3e, f, g). The sequence variants detected in affected members were present in heterozygous state in obligate carriers of the families. To exclude the possibility that the splice site mutation identified in the family F does not represents a neutral polymorphism in the Pakistani population, a panel of 100 ethnically matched, unrelated unaffected control individuals were screened directly by sequencing. Non-polymorphic nature of the missense mutation identified in five families was verified by screening 250 unrelated, unaffected control individuals.

## Discussion

Six consanguineous Pakistani families (A-F), exhibiting typical features of Acromesomelic Dysplasia, type Maroteaux (AMDM), were investigated in the present study. Five of these families (A-E) were probably distantly related. Diagnosis of AMDM in the six families was based upon characteristic physical and x-ray findings in the affected individuals. Physical and clinical features observed in affected members of the six families were similar to those reported earlier in several families with AMDM of different ethnic origin [3,4,15,16]. Affected individuals of family F had long faces, a feature not observed in five other families, presented here. An affected individual with a long face was also reported by Hachiya et al. [16] in a Japanese patient. The heterozygous carriers in all the present six families were shorter in statures. Findings of the reduced statures of the heterozygous carriers were reported earlier in other families with AMDM [4,15]. Tamura et al. [6] observed reduced stature in mouse heterozygous for the NPR-B mutation. The shorter stature found in heterozygous carriers is probably caused by a dominant-negative effect of the mutant allele as suggested by Hachiya et al. [16].

Genotyping analysis in the six families established linkage to the gene *NPR2*, mapped earlier on chromosome 9p13-q12 [3,4]. DNA sequence analysis of the gene in six families (A-F) identified two novel disease causing sequence variants. In five families (A-E), a novel homozygous missense mutation (c.2720 C > T; p.T907M) was detected in all sixteen affected individuals. In family F, sequence analysis identified a novel splice donor site mutation in intron 20 of the gene (c.2986 + 2 T > G). To date, 23 mutations in the gene *NPR2* have been reported in families with different ethnic background from around the world [4,15,16]. This included 4 nonsense, 5 frameshift, 2 splice site and 12 missense mutations in 23 families with AMDM reported.

The human *NPR2* gene spans about 16.5 kb, contains 22 exons encoding 1047 amino acids protein [3]. Potter [17] has shown that guanylyl cyclase-linked natriuretic peptide receptors consist of 450 amino acids extracellular ligand-binding domain, 20 amino acids of single hydrophobic transmembrane domain and 570 amino acids of intracellular domain. Intracellular domain contains 3 subdomains including a kinase homology domain of 250–260 amino acids, a coiled-coil dimerization domain of 40 amino acids, and carboxyl-terminal guanylyl cyclase catalytic domain of 250 amino acids [18].

A homozygous missense mutation (p.T907M), identified in five families, in the present study, is lying in the guanylyl cyclase domain of the NPR-B*.* The threonine residue at position 907 is highly conserved among various species. Analysis of the protein sequence by protein prediction tool PolyPhen (http://genetics.bwh.harvard.edu/pph/) revealed that the substitution of polar threonine by a non-polar amino acid methionine (p.T907M) could potentially have a damaging effect on NPR2 structure with a PSIC (Position specific independent counts) score of 1.00. Hume et al. [19] hypothesized that missense mutations in NPR-B, resulting in AMDM, is primarily due to arrest of the receptor trafficking in the endoplasmic reticulum (ER). Therefore, it is more likely that the missense mutation (p.T907M), identified here, also results in retention of the mutant NPR-B in the ER and has become non-functional.

The splice site mutation (c.2986 + 2 T > G), identified in one family here, can cause skipping of exon 20, as well as abolish most of the c-terminal guanylyl cyclase domain of NPR2 protein. This leads to synthesis of truncated or non-functional protein, possibly due to nonsense-mediated mRNA decay (NMRD) [20].

At least two studies have shown that mutations in GC-binding domain in the gene Npr2 result in impaired endochondral ossification and severe dwarfism in mice [6,21]. Since NPR-B mediate its biological function through GC domain, it is highly likely that the effect on the structure of NPR-B will affect the natriuretic peptide-dependent physiological response occurs through synthesis of cGMP. This in turns will affect the cGMP signaling effects which occurs through cGMP binding proteins including cGMP dependent protein kinase (PKG), cGMP binding phosphodiesterases (PDEs) and cyclic nucleotide-gated ion channels. The best studied cGMP signaling effects occurs through PKGs. Deletion of a membrane bound PKGII, which has been found in high concentration in brain, chondrocytes and bones [22], results in dwarfism in rodents [22,23]. The genetic data pertaining to mutations identified in Nppc and Npr-b in mice and NPR-B in human clearly showed the involvement of CNP/NPR-B/cGMP signaling pathway in bone development.

## Conclusions

We have reported two novel mutations in the gene *NPR2*, which result in Acromesomelic Dysplasia, type Maroteaux (AMDM). The presence of the same mutation (p.T907M) and haplotype in five families (A, B, C, D, E) is suggestive of a founder effect. The study, presented here, confirm the significant role assigned to NPR2 in development of bones in human.

## Consent

Patients and their guardians provide written consent for publishing photographs and other material.

## Competing interests

The authors declare that they have no competing interests.

## Authors’ contributions

SK and RHA participated in the design of the study, performed molecular testing and manuscript writing. SA, MN, NM studied families, collected blood samples and extracted DNA. WA analyzed the data, participated in manuscript preparation and collected funds for the study. All authors read and approved the final manuscript.

## Pre-publication history

The pre-publication history for this paper can be accessed here:

http://www.biomedcentral.com/1471-2350/13/44/prepub
